# The Brisbane Systems Genetics Study: Genetical Genomics Meets Complex Trait Genetics

**DOI:** 10.1371/journal.pone.0035430

**Published:** 2012-04-26

**Authors:** Joseph E. Powell, Anjali K. Henders, Allan F. McRae, Anthony Caracella, Sara Smith, Margaret J. Wright, John B. Whitfield, Emmanouil T. Dermitzakis, Nicholas G. Martin, Peter M. Visscher, Grant W. Montgomery

**Affiliations:** 1 University of Queensland Diamantina Institute, University of Queensland, Princess Alexandra Hospital, Brisbane, Queensland, Australia; 2 Queensland Institute of Medical Research, Herston, Brisbane, Australia; 3 Department of Genetic Medicine and Development, University of Geneva Medical School, Geneva, Switzerland; 4 The Queensland Brain Institute, The University of Queensland, Brisbane, Queensland, Australia; Johns Hopkins University, United States of America

## Abstract

There is growing evidence that genetic risk factors for common disease are caused by hereditary changes of gene regulation acting in complex pathways. Clearly understanding the molecular genetic relationships between genetic control of gene expression and its effect on complex diseases is essential. Here we describe the Brisbane Systems Genetics Study (BSGS), a family-based study that will be used to elucidate the genetic factors affecting gene expression and the role of gene regulation in mediating endophenotypes and complex diseases.

BSGS comprises of a total of 962 individuals from 314 families, for which we have high-density genotype, gene expression and phenotypic data. Families consist of combinations of both monozygotic and dizygotic twin pairs, their siblings, and, for 72 families, both parents. A significant advantage of the inclusion of parents is improved power to disentangle environmental, additive genetic and non-additive genetic effects of gene expression and measured phenotypes. Furthermore, it allows for the estimation of parent-of-origin effects, something that has not previously been systematically investigated in human genetical genomics studies. Measured phenotypes available within the BSGS include blood phenotypes and biochemical traits measured from components of the tissue sample in which transcription levels are determined, providing an ideal test case for systems genetics approaches.

We report results from an expression quantitative trait loci (eQTL) analysis using 862 individuals from BSGS to test for associations between expression levels of 17,926 probes and 528,509 SNP genotypes. At a study wide significance level approximately 15,000 associations were observed between expression levels and SNP genotypes. These associations corresponded to a total of 2,081 expression quantitative trait loci (eQTL) involving 1,503 probes. The majority of identified eQTL (87%) were located within *cis*-regions.

## Introduction

Dissection of the genetic architecture underlying quantitative traits and complex disease is essential to our understanding of the aetiology of complex diseases that cause most of the disease burden in society. Genome-wide association studies (GWAS) have proven to be an effective tool for identifying common causal loci of moderate to large effect size associated with human diseases and traits [1], [2]. However, as has been well discussed, GWAS have struggled to discover loci that collectively explain large proportions of the heritability of most complex traits [3], [4]. It has been proposed [5]–[7] that the nature of genetic variance for complex disease may be different to that of Mendelian disease that are caused by protein coding mutations, in that it may result from hereditary changes in gene regulation rather than gene variants that alter protein function. A number of recently published studies support this hypothesis [8]–[10]. More specifically, genetic differences between individuals in quantitative traits, endophenotypes (phenotypes that are risk factors for disease) and susceptibility to common diseases may be caused by differences in gene expression at a number of interacting loci [11]. Therefore, understanding the genetic basis of gene expression is likely to lead to a better understanding of genetic variation of quantitative traits and risk factors for common diseases.

Transcript abundance is a proximal endophenotype affected by genetic factors and has already facilitated the identification of candidate susceptibility genes for metabolic disease traits [12], psychiatric disorders [13], [14], asthma [15] and Crohn's disease [16]. This has mostly been possible when the tissue of expression was relevant to the interrogated complex trait, as disease phenotypes tend to manifest themselves only in certain tissues. Common disease is an endpoint of a complex pathway and there are likely to be many possible perturbations, or combinations thereof, in the pathways that lead to disease. Understanding the pathway as a whole, including the genetic determination of major hubs of gene regulation and how they relate to endophenotypes, will therefore be a major step in understanding the ultimate disease outcome and may provide information on the optimal choice of where to intervene in the pathway to prevent or treat disease.

Here we outline a major family-based system genetics study initiated to enhance our knowledge about common trait susceptibility by providing genome-wide expression and genotype data for 962 extensively phenotyped individuals in a family based design. This study builds on existing genotype and phenotype data collected at Queensland Institute of Medical Research (QIMR) through the collection of gene expression levels in whole blood (WB) (on all individuals) and lymphoblastoid cell lines (LCL) (on a subset of 50 MZ pairs). This study will help illuminate the genetic parameters underlying regulation of gene expression and to understand the genetic basis of complex traits through the relationship between gene expression and endophenotypes. We present a detailed description of the BSGS and report results on the association of expression levels with SNP genotypes from 862 BSGS individuals.

### BSGS resource

**Figure 1:**
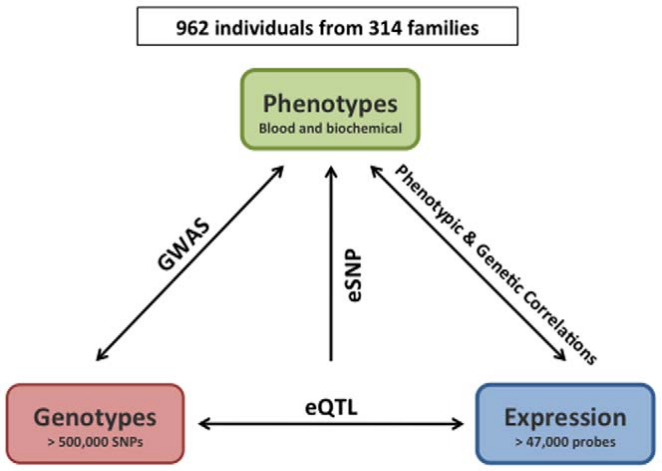
Summary of BSGS study design. The structure of the study design allows us to investigate fundamental questions about the genetic basis of gene expression and their correlation with phenotypes that are known risk factors for disease.

BSGS comprises genotypic, expression and phenotype data that can be used to elucidate the genetic basis of gene expression and the relationship between gene expression and complex phenotypes. BSGS and its rationale are summarised in figure 1.

**Figure 2:**
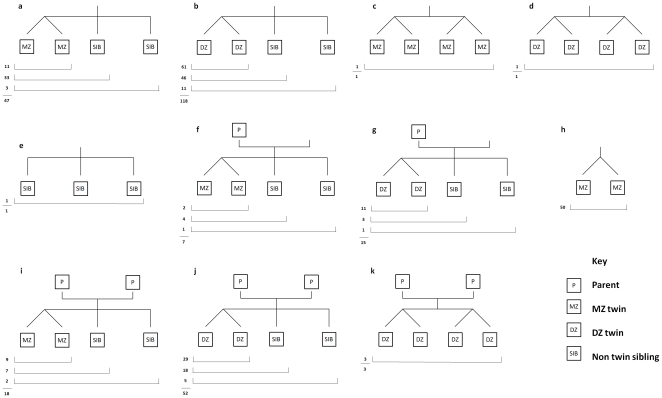
Samples collected in BSGS comprise of a number of different families. Family structure **h** represents the 50 MZ pairs comprising the stage I study. The remaining family structures are from stage II. The numbers of each family structure are given below the pedigree diagram. By utilising expression information contained between and within twin pairs, siblings and between progeny and parents we are able to estimate genetic and non-genetic variance components using linear mixed models.

Gene expression data were generated for a total of 962 individuals from 314 families, for which we have genotype and phenotype data. Of the 962 individuals there are 128 MZ pairs (68 female and 60 male) and 206 DZ pairs (51 female, 53 male and 102 opposite sex). For 72 families expression data were collected on both parents as well as their offspring, allowing us to test parent-of-origin effects for gene expression. The 314 families comprise a variety of family structures and sizes, details of which are given in Figure 2. Details of the pairwise relationships are given in Table 1.

**Table 1:** Relationship pairing between 962 individuals in BSGS.

Relationship pairs	Code	N	Notes
Monozygotic twins	MZ	128	68 female pairs; 60 male pairs; 50 MZ pairs form stage I, where we have expression data from both WB and LCL RNA sources
Dizygotic twins	DZ	206	51 female pairs; 53 male pairs; 102 mixed sex pairs
Siblings	SIB	343	81 female pairs; 82 male pairs; 180 mixed sex pairs
Parent – Offspring	PO	425	98 father – daughter pairs; 103 father – son pairs; 113 mother – daughter pairs; 111 mother – son pairs
Parent – Parent	PP	71	

BSGS comprises the following data;

#### Expression

Gene expression data were collected in two stages: **Stage I** - involving 50 MZ pairs, on whom expression data were collected for both WB and LCL; **Stage II** - the remaining 862 individuals, where expression data were collected in WB only. A full description of the data collection for the stage I study is given in Powell et al. [17], and so for the sake of brevity, we focus on the description of stage II here. Transcript expression levels in WB were measured with over 47,000 genome-wide probes using the Illumina HT-12 v4.0 microarray chip (see materials and methods). Of the probes on the HT-12 v4.0 chip, approximately 29,000 are well characterized and annotated coding transcripts, 11,000 are coding transcript but poorly annotated, 4,000 are non-coding and 3,000 are experimentally confirmed mRNA sequences that align to EST clusters.

#### Genotypic

Individuals are genotyped using the Illumina 610K chip [18]–[20]. After quality control procedures (see materials and methods) over 500 k genome-wide SNPs are available.

#### Phenotypes

A large number of hematology and blood biochemical phenotypes were measured at time of sample collection and later studies [19], [20]–[25]. In many cases these phenotypes have been analysed as part of a larger QIMR twin study focusing on linkage, association and heritability analyses. A brief summary of published results and phenotypes is given in table 2. Of particular relevance are the phenotypes measured in blood samples, including biochemical measures, because of their close relationship to the tissue in which expression levels have been measured. Such a relationship provides an ideal test case for the systems genetics approach that we are taking.

**Table 2:** Central phenotypes in BSGS and a brief summary of previous studies identifying genetic parameters and association signals.

Phenotypes	Summary	Reference
Hemoglobin concentration	Hemoglobin phenotypes have been associated with SNPs in *TMPRSS6, LRRC16A, HK1* and *HK2*.	[19], [20]
Red blood cell count	Association with SNPs close to *IRX6*.	[20]
Platelet count	Platelet count has suggestive association with SNPs in *KCNIP*.	[20]
White blood cell count	White blood cell count has suggestive association with SNPs in *MACF1*.	[20]
Monocytes	Monocyte count is associated with SNPs close to *BAG4* and *ITGA4*.	[20]
Eosinophils	Eosinophil count has suggestive association with SNPs in *ITPR1*.	[20]
CD4+/CD8+ T-cell ratio	Collectively, these phenotypes are associated with SNPs in the MHC and the Schlafen family of genes. They are also endophenotypes for Type 1 Diabetes, HIV-1 immune control and autoimmune diseases.	[25]
Plasma Cholesterol (HDL and LDL) and Triglyceride concentrations	A known endophenotypes for cardiovascular disease. Data comprising part of BSGS have shown strong associations between Cholesterol and genes on chromosome 19 and between Triglyceride and genes on chromosome 7.	[18], [22]
Blood pressure	An important endophenotype for hypertension. Data comprising part of BSGS have shown strong associations between blood pressure and genes on chromosomes 4,5,14 and 17.	[23], [24]
Iron, Ferritin and Transferrin levels	Collectively, these phenotypes show association with SNPs in *TMPRSS6*, *HFE, PGM1* and *TF*.	[19], [61], [62]

## Results

Here we present results on expression data quality and an eQTL analysis using expression data from WB for the 862 (Stage II) individuals in BSGS. Details of expression quality for stage I individuals are given in Powell et al. [17].

### Stage II expression data quality

Gene expression levels were generated for the 862 individuals from WB samples using the PAXgene™ tube system. Expression levels of extracted mRNA were measured using Illumina HumanHT-12 v4.0 whole genome chip. For use as a research resource the quality of the expression data is important, although quality control procedures are likely to differ depending on specific analyses. Original data consists of raw, un-normalised, expression levels with chip background levels subtracted for 47,323 probes. Illumina software GenomeStudio also calculates a detection *p*-value (see methods), which represents the probability that a given transcript is expressed above the chip background level. A value below a *p*-value threshold (i.e. 0.05) indicates a gene is detected and thus, the number of detected transcripts is a good overall indicator of sample expression quality, with all samples on a given chip expected to have similar numbers of transcripts detected. All 862 samples show high numbers of probes detected with *p*<0.05 (mean = 17,310 and SD = 1,512) with similar numbers within and between chips (Figure S1). These levels are similar or slightly higher than other published reports using Illumina chips (for example in Monocytes [26], Lymphoblastoid cell lines (LCL) [27], [28], Fibroblasts and T-cells [28]).

Gene expression is known to vary between individuals due to differences in environmental [29], [30] and genetic [31] factors. Additionally, differences in the genetic control of gene expression between tissues [17], [32], coupled with specific environments of cell types, lead to certain genes being expressed in some tissues and not others at the time of sample collection [33]. A consequence is that we expect many probes not to be expressed in a proportion of the individuals in our sample. Studies of gene expression data use a variety of criteria to select probes for inclusion in analyses. A commonly used criterion is that a given gene must be detected as expressed in a certain proportion of the samples, with the particular proportion decided based on requirements and constraints of the analyses. Of the 47,323 probes 5,364 (11.3%) are not detected as expressed in any of the 862 individuals, whilst 6,281 (13.3%) are detected as expressed in all individuals (Figure S2).

### Identification of eQTL

Of the 47,323 probes whose expression levels were measured on the chip, only probes that were well characterised and detected as expressed in 10% of the sample were carried forward, leaving a total of 17,926 probes (corresponding to 13,533 RefSeq genes) whose expression was tested for association with the 528,509 SNPs. At the study wide significance threshold (5.25×10^−12^) a total of 14,916 associations were identified involving 10,421 SNPs (eSNPs) and 1,503 probes. In situations where a probe had multiple significant SNP associations, independent eQTL were called if the distance between significant SNPs was greater than 2MB. Using this criterion a total of 2,081 eQTL were called for the 1,503 probes with 1,357 (90%) of probes having a single eQTL and 19 probes with greater than five eQTL. The median number of significant SNPs within an eQTL region was 4, although 351 (16.8%) of eQTL contained over 10 significant SNPs and 27 (1.3%) over 50. Of the 10,421 eSNPs the majority (73.6%) were associated with a single probe whilst the remaining 26.4% have significant associations with two or more probes, with the maximum being eight.

**Figure 3:**
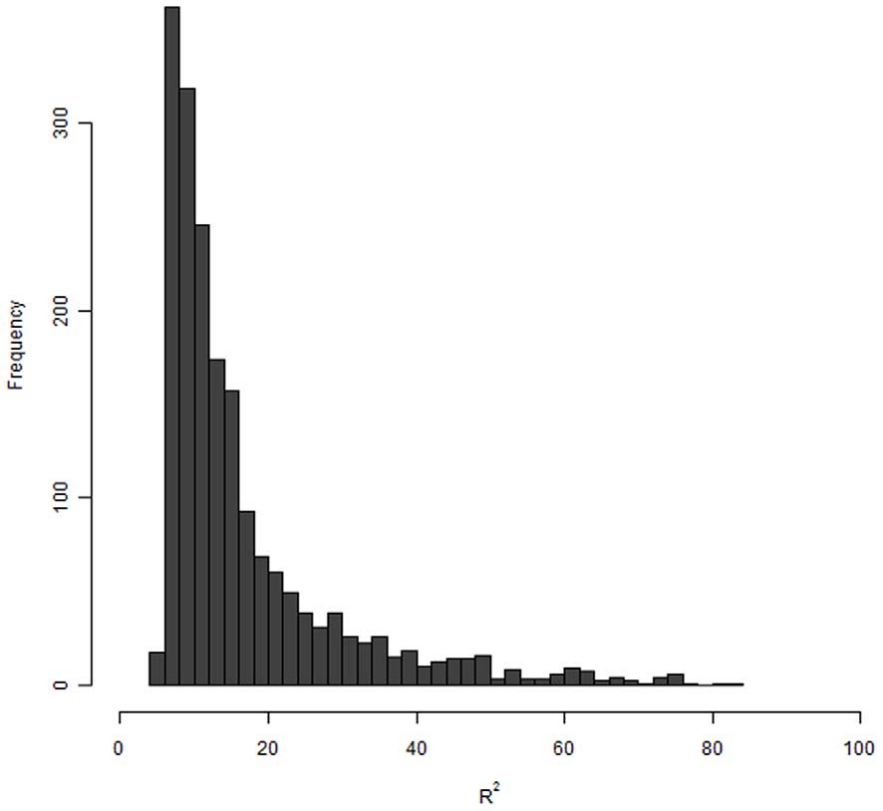
Distribution of the *R^2^* observed for the best eSNP from the 1,885 eQTLs.

Among the 2,081 eQTL the SNP with the smallest *p*-value had *R^2^* (proportion of expression variability explained) from 4.6% to >80% with a median of 12.1% (Figure 3). A total of 328 eQTLs had *R^2^* greater than 25% and 61 greater than 50%. It is worth noting that these estimates will be biased upwards because hypothesis testing and estimation were performed on the same data (“winner's curse”).

**Figure 4:**
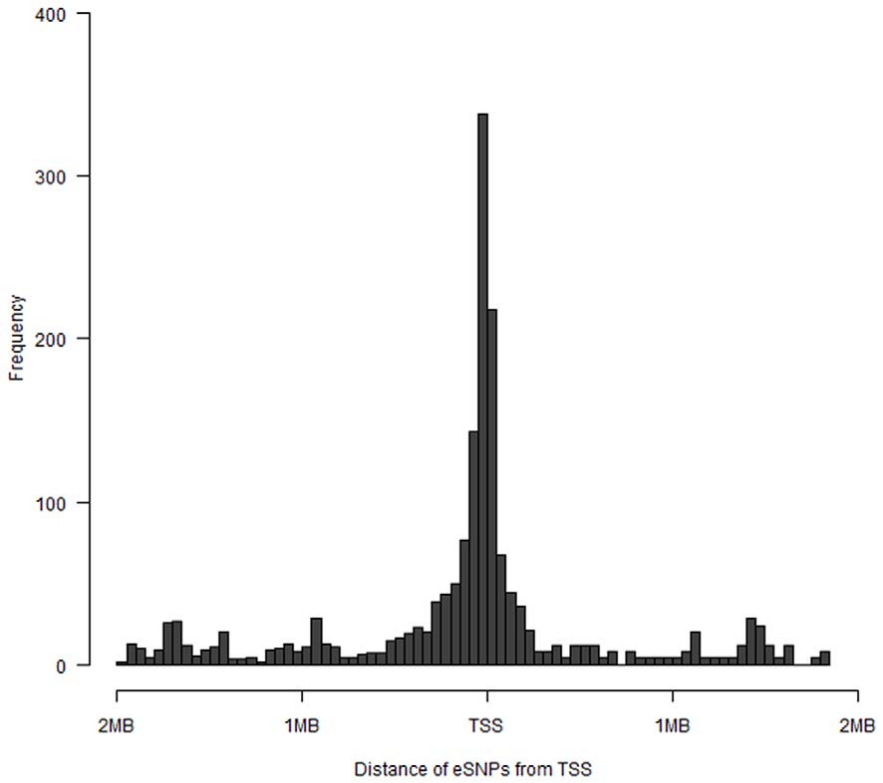
The distribution of *cis*-eSNPs distance from the Transcription Start Site (TSS). The distances of eSNPs from the TSS were divided into 50KB bins across the *cis*-region.

Of the 2,081 eQTL, 1,810 (87%) are *cis*-acting, being located within a 2MB region either side of the 3′ or 5′ end of the Transcription Start Site (TSS). The remaining 271 eQTL are *trans*-acting, although the majority of these (232) are located on the same chromosome as their probes TSS. Twelve probes had both *cis*-and *trans*-acting eQTL. To explore the location of *cis*-eQTLs, the distance of the most significant SNP for each *cis*-eQTL per probe was mapped relative to the TSS. In agreement with previous studies [28], [34]–[35] a strong signal was found close to the TSS, with no discernable trend in a 3′ or 5′ direction (Figure 4). This symmetrical trend is likely to reflect variation in core regulatory sequences such as promoter elements. Details of the top twelve *cis*-eQTL (based on eSNP *p*-values) are given in table 3.

**Table 3:** Top *cis*-eQTL results.

Probe ID	Gene	Probe Chromosome	TSS bp	Top SNP	Top SNP location	-log10 *p*-value	*R^2^*
ILMN_1715169	HLA-DRB1	6	32654825	rs9271170	6 - 32577889	131.5	73.5
ILMN_1743145	ERAP2	5	96274820	rs10051637	5 - 96279490	131.0	81.6
ILMN_1798177	CHURC1	14	64471249	rs7143432	14 - 65379146	130.4	83.0
ILMN_2209027	RPS26	12	54722494	rs10876864	12 - 56401085	122.0	74.6
ILMN_2403228	CLEC12A	12	10029119	rs7313235	12 - 10132283	121.3	75.8
ILMN_2352023	RIPK5	1	203378372	rs12139373	1 - 205054879	114.4	70.2
ILMN_2312606	IRF5	7	127973722	rs6965542	7 - 128655918	112.6	74.8
ILMN_1791511	TMEM176A	7	150133038	rs7806458	7 - 150476888	107.5	66.4
ILMN_2038775	TUBB2A	6	3154070	Rs9392465	6 - 3162378	107.2	66.7
ILMN_3298167	ZSWIM7	17	15879944	rs1045599	17 - 15879910	105.5	65.9
ILMN_1661266	HLA-DQB1	6	32736001	rs9273349	6 - 32625869	102.0	61.2
ILMN_2313901	PAM	5	102340879	rs28092	5 - 102149795	99.7	66.2

The chromosome and base pair position of the probe transcription start site (TSS) are given for each probe. *R^2^* is the proportion of transcript level variance explained by the SNP with the strongest association.

**Figure 5:**
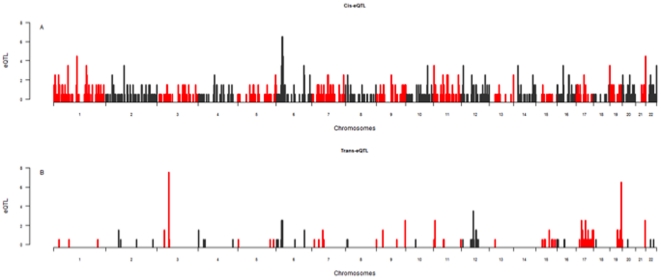
Positions of *cis* (A) and *trans* (defined as greater than 2MB from the transcription start site) (B) eSNP across the genome. The number of eSNP within 1MB bins is shown. A single eSNP represents a unique eQTL.

Regulatory control for gene expression, particularly *trans*-acting, is not evenly distributed across the genome [36]. To provide an outline of the positions of regulatory control for gene expression across the genome, we produced a Manhattan plot showing the number of eSNP (each eSNP represents a single eQTL) within each 1MB region (Figure 5). *Cis*-acting eSNPs are distributed across the genome, as has been reported by multiple studies [26]–[27], [37]. We observe that *trans*-acting eSNPs are often found in close proximity to one another, supporting the concept of *trans*-regulatory hotspots [38].

Many studies mapping eQTL consider just associations within the *cis*-region: the main benefit being a considerably reduced burden of multiple testing compared to studies including *trans*-acting SNPs. Given correlations exist between both SNP genotypes and probe expression levels, it is likely that our study-wide significance threshold (corrected for all SNP by probe tests) results in true associations being missed. By reducing the significance threshold to less conservative levels, the number of *trans*-eQTL considerably increased, as expected by chance (Table 4). However, the number of *cis*-eQTL only moderately increased, indicating that conservative threshold used did not result in a considerable under estimation of the true number of *cis*-eQTL (Table 4).

**Table 4:** Number of associations and eQTL at various levels of significance threshold.

Significance threshold	Total associations	Expected number of associations	Total significant SNPs	Probes with 1+ significant SNP	eQTL	Cis-eQTL	Trans-eQTL
10e^−8^	31,032	951	20,068	3,969	5,679	2,673	3,006
10e^−10^	20,049	9.5	13,319	1,933	2,512	1,953	559
**5.25e^−12^**	**14,210**	**0.05**	**10,160**	**1,503**	**1,885**	**1,529**	**256**
10e^−15^	10,519	0	7,468	1,126	1,507	1,256	251
10e^−25^	4,362	0	3,382	572	731	639	92
10e^−50^	896	0	764	185	199	172	27
>10e^−100^	60	0	55	27	27	22	5

The study-wide significance threshold employed for our eQTL analysis is highlighted. The expected number of associations is the number of association that are expected to be observed under the null hypothesis of no associations between probe expression levels and SNPs.

## Discussion

Through understanding the genetic control of gene expression and its relationship with indicators of common disease we can gain a better understanding of disease itself. Common disease is an endpoint of a complex pathway of genetic and environmental factors and there are likely to be many possible perturbations in the pathways that lead to disease. Gaining insights into the pathway as a whole, including the genetic control of major hubs of gene regulation and how they relate to endophenotypes, is a major step in understanding the ultimate outcome of (disease) and may provide information on the optimum choice of where to intervene in the pathway to prevent or treat disease. Recent success at identifying genetic determinants for networks of regulatory control affecting metabolic disease [39] has highlighted the application of systems genetic approaches in understanding the aetiology of complex phenotypes. We aim to use BSGS for the identification of polymorphisms, or combinations thereof, that affect gene regulation in one or more genes which in turn cause differences between individuals in endophenotypes or susceptibility to disease and may lead to drug discovery or other treatments to decrease the burden of common disease in society.

The cellular origin of many biochemical and blood phenotypes is often complex with some traits originating in other tissues but displayed in blood. For example, insulin originates from β-cells in the pancreas but is carried in the blood where is performs its role of regulating glucose levels. However, insulin levels are regulated by multiple factors, many relating to blood components and so it is reasonable to expect that the expression of some genes in blood will have an effect on insulin levels. Thus, using a tissue closely related to phenotypes we are able to relate transcript abundance to the blood and biochemical traits and hopefully expound the role of gene regulation in mediating endophenotypes and complex disease. Nevertheless, findings should be analysed with caution as we and others [17], [32], have recently shown that the genetic basis for gene expression is often different between tissues.

We hypothesise that the indirect route from genotype to phenotype via gene expression is more informative to elucidate the nature of complex trait variation than the direct route from genotype to phenotype. The expression data collected from families allows us to estimate quantitative genetic variance components through the use of linear mixed models and estimation methods such as restricted maximum likelihood (REML) [40]. By utilising information contained between and within twin pairs, siblings and between progeny and parents we are able to estimate additive and non-additive genetic effects of gene expression and can explicitly test a variance partition model that includes parent-of-origin effects.

Here we have presented results of an eQTL study involving ∼18 k probes, each tested against >500 k SNPs. Despite numerous large scale studies reporting eQTL [26]–[28], [34], [37], [41]–[42], generating eQTL results for a particular study is important as differences in the genetic control of gene expression between populations [43]–[45] and between tissues [17], [32] is well known. Furthermore, here, and elsewhere [26], [41]–[42] large sample sizes have permitted the investigation of *trans*-acting eSNP often ignored by smaller studies [28], [34].

## Methods

### Sample Collection

Individuals present in this study were recruited as part of the Brisbane Twin Nevus and cognition studies (known as BTN and MAPS respectively). This study was approved by the Queensland Institute for Medical Research-Human Research Ethics Committee. All participants gave informed written consent. As described in detail elsewhere [21], [46]–[50], adolescent MZ and DZ twins, their siblings, and their parents have been recruited over a 16 year period into an ongoing study of the genetic and environmental factors influencing pigmented nevi and the associated risk of developing skin cancer and cognition. The sample is of northern European origin (mainly Anglo-Celtic). A Principal Component Analysis (PCA) comparing individuals in this study to HapMap3 [51] and GenomEUtwin [52] populations shows the close similarity of ancestry to northern European populations (Figure S3). All participants gave informed consent, and the study protocol was approved by the appropriate institutional review boards.

### Analysis of Stage II data

#### Genotyping

DNA samples from each individual were genotyped by the Scientific Services Division at deCODE Genetics, Iceland, using the Illumina 610-Quad Beadchip. Genotypes were called with the Illumina BeadStudio software. Full details of genotyping procedures are given in Medland et al. [53]. Standard QC filters were applied so that further analysis was on samples and SNPs with high data quality. We first applied filters to SNP data before evaluating genotyping quality per individual. After removing SNPs with minor allele frequencies (MAF) <1% with a mean BeadStudio GenCall <0.7 a total of 528,509 remained for further analysis.

#### Gene expression quantification

Whole blood for expression profiling was collected directly into PAXgene™ tube (QIAGEN, Valencia, CA). Total RNA was extracted from PAXgene™ tubes using the WB gene RNA purification kit (QIAGEN, Valencia, CA). RNA from all samples was run on an Agilent Bioanalyzer to assess quality and to estimate RNA concentrations RNA was converted to cDNA, amplified and purified using the Ambion Illumina TotalPrep RNA Amplification Kit (Ambion).

Expression profiles were generated by hybridising 750 ng of cRNA to Illumina HumanHT-12 v4.0 Beadchip according to Illumina whole-genome gene expression direct hybridization assay guide (Illumina Inc, San Diego, USA). Briefly, 500 ng of total RNA were used to generate biotinylated cRNA, which was fragmented and hybridised to an Illumina whole genome expression chip, HumanHT-12 v4.0 for 18 h at 58°C. Beadchips were then washed and stained and subsequently scanned to obtain fluorescence intensities. Samples were scanned using an Illumina Bead Array Reader. Samples were randomised across chips and chip positions, with check for balance across families, sex and generation.

### eQTL analysis

#### Normalisation and processing

The following normalisation procedures were applied to the raw expression data for the eQTL analysis. Pre-processing of data generated by the Illumina Bead Array Reader was done using Illumina software, GenomeStudio (Illumina Inc., San Diego). Pre-processing included; correction for chip background effects, removal of outlier beads, computation of average bead signal and calculation of detection *p*-values using negative controls present on the array. Removal of chip background effects can lead to negative expression levels for transcripts with low levels of measured expression. To avoid problems with further normalisation procedures, negative values were changed to missing data identifiers. Thus, in subsequent normalisation procedures and analyses probes coded as missing are ignored.

The Illumina HT-12 v4.0 chip contains 47,323 probes, although some probes are not assigned to RefSeq genes. To avoid spurious associations, we removed 678 probes for which at least one HapMap 3 (NCBI build 36, dbSNP b126) SNP was within the probe sequence [54]. For the eQTL analysis we removed any probes where less than 10% of samples had a detection *p*-value<0.05. Of the 24,317 probes retained, the mean of the proportion of samples with *p*-values<0.05 was 97%, implying that relatively little missing data remained within the expression dataset. After removing 6,322 putative and/or not well-characterised genes i.e. probe names starting with HS (*n* = 1,841), KIAA (*n* = 158) and LOC (*n* = 4,323), 17,926 well-characterised detected probes remained for analysis, which corresponds to 13,486 RefSeq genes.

To minimise the influence of overall signal levels, which may reflect RNA quantity and quality rather than a true biological difference between individuals, the following standardisation procedures were applied to the 17,926 probes. Adjusted expression levels for each probe were transformed using a quantile transformation [55], [56] to achieve a stabilized distribution across average expression levels. Further normalisation was performed to allow expression levels to be compared across chips and genes. This was achieved fitting a linear mixed model;
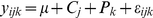
(1)Where 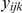 is the log-transformed expression level for individual *i* on chip *j*. The variable 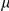 represents the mean expression level across all individuals and 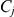 and 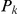 are random effects removing variation in the data due to chip *j* and chip position *k* respectively, and 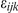 is the residual. The between chip variance is expected to be small due to the scaling that was performed during the pre-processing of the data. The residuals from this model were used in all further analyses. To increase robustness, the distribution of normalised expression levels for each probes were tested for deviation from normality using the Shapiro-Wilk test. All 17,926 probes had normally distributed (*p*<0.05) expression levels.

#### Testing for association

We tested for association between the 528,509 genotyped SNPs and the normalised expression levels of the 17,926 probes using the FASTASSOC component of MERLIN [57], [58]. The FASTASSOC option fits a simple linear regression model to estimate an additive effect for each probe and SNP combination, with SNP genotypes coded as the number of copies of the minor allele (0, 1 or 2) carried by each individual. We used the Lander-Green algorithm [58], [59], implemented in Merlin, to estimate expected genotype scores for individuals with missing genotype data. Covariates of sex and generation were included in the model, where generation denotes either the parental or the adolescent generation. Previous analysis has shown (not published) that generation is a useful substitute for age without the burden of additional degrees of freedom. The model applies a variance component approach to account for the correlations between different expression levels within each family. The model fit is evaluated using a score test, which substantially reduces computational time compared to maximum-likelihood methods, at the expense of a slight loss of power [58].

Conditional regression analysis was used to address the potential to miss secondary eQTL in linkage disequilibrium (LD) with other eQTL. For each probe with an identified eQTL we corrected for the main effects of the top eSNP (SNP with the largest *R^2^*) by regressing its genotypes against the expression levels. Residuals from this analysis were then used for second round of eQTL mapping, allowing us to detect independent eQTL. If additional eQTL were identified from this second round of analysis, the process was repeated, correcting for the main effects of the top eSNP from the first and second eQTL using multivariate regression.

Associations were evaluated in two categories depending on the location of the SNP relative to the transcription start site (TSS). Cis-eQTL were defined as associations between SNPs within 2MB of either the 3′ or 5′ end of the TSS. We defined *trans*-associations as associations involving SNPs elsewhere in the genome. To correct for multiple testing, we used a study-wide significance level of 0.05, corrected for the number of SNP by probe associations tested, corresponding to a *p*-value threshold of 5.25×10^−12^.

We tested for the effects of population structure and cryptic relatedness between individuals by applying the method ‘genomic control’ [60] to results of the association analysis. We derived a coefficient of 1.002, indicating negligible population stratification.

## Supporting Information

Figure S1 **GenomeStudio provides a** ***p*** **-value for each transcript in each sample.** For a given sample the number of transcripts with *p*-values below a given threshold provides an indication of its quality. Figure S1 shows the number of transcripts with *p*<0.05 for each sample with each colour representing a single chip.
(DOCX)

Figure S2 **The distribution of the number of probes detected as expressed in stage II.** A total of 47,323 are measured on the Illumina HT-12 v4.0 chip, of these 5,364 (11.3%) are not detected as expressed in any of the individuals, whilst 6,281 (13.3%) are detected as expressed in all individuals.
(DOCX)

Figure S3 **A Principle Component Analysis (PCA) of 16 global populations and the individuals collected in this study.** Principal Component one (PC1) and two (PC2) values were derived from approximately 280,000 autosomal markers. Populations samples marked with *1 were collected as part of the HapHap3 project [1] and *2 as part of the GenomEUtwin project [2]. **ASW** ^*1^ African Americans, **CEU** ^*1^ European Americans, **CHB** ^*1^ Han Chinese, **CHD** ^*1^ Chinese, **GIH** ^*1^ Guajarati-Indians, **JPT** ^*1^ Japanese, **LWK** ^*1^ Luhya Kenyans, **MEX** ^*1^ Mexicans, **MKK** ^*1^ Maasai Kenyans, **TSI** ^*1^ Italians, **TRI** ^*1^ Yorubans Nigeria, **DEN** ^*2^ Danish, **FIN** ^*2^ Finish, **NET** ^*2^ Dutch, **SWE** ^*2^ Swedish, **UK** ^*2^ British.
(DOCX)
