# Supplementary material for: The Brisbane Systems Genetics Study: Genetical Genomics Meets Complex Trait Genetics
Source: PLoS One. 2012 Apr 26;7(4):e35430. doi: 10.1371/journal.pone.0035430 (PMC3338511; doi:10.1371/journal.pone.0035430)
Supplement: Figure S1 — GenomeStudio provides a p -value for each transcript in each sample. For a given sample the number of transcripts with p-values below a given threshold provides an indication of its quality. Figure S1 shows the number of transcripts with p<0.05 for each sample with each colour representing a single chip. (DOCX) [file pone.0035430.s001.docx]

**Figure S1**


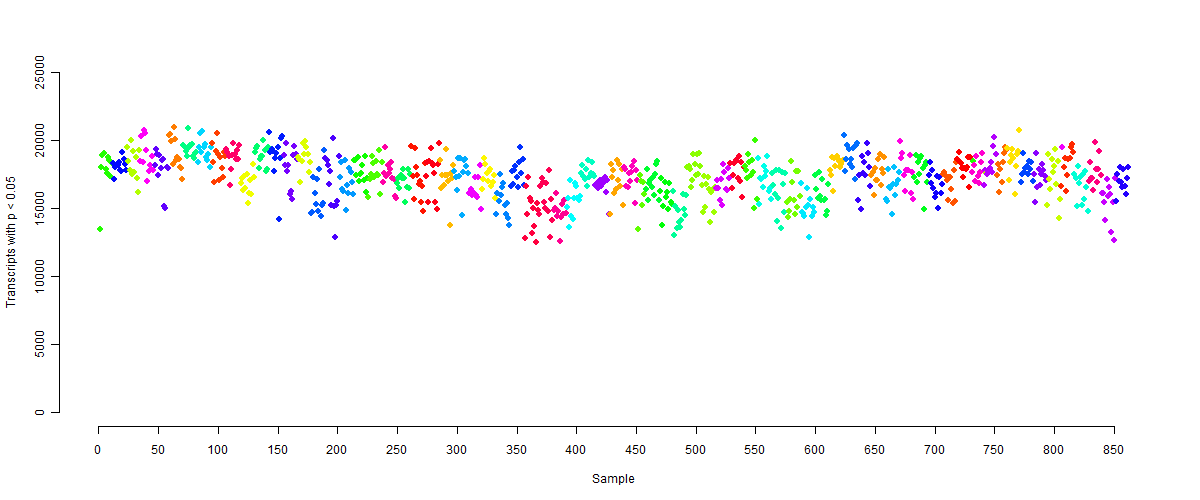


**Figure S1 |** GenomeStudio provides a *p*-value for each transcript in each sample. For a given sample the number of transcripts with *p*-values below a given threshold provides an indication of its quality. Figure S1 shows the number of transcripts with *p* < 0.05 for each sample with each colour representing a single chip.
