# Supplementary material for: The Brisbane Systems Genetics Study: Genetical Genomics Meets Complex Trait Genetics
Source: PLoS One. 2012 Apr 26;7(4):e35430. doi: 10.1371/journal.pone.0035430 (PMC3338511; doi:10.1371/journal.pone.0035430)
Supplement: Figure S2 — The distribution of the number of probes detected as expressed in stage II. A total of 47,323 are measured on the Illumina HT-12 v4.0 chip, of these 5,364 (11.3%) are not detected as expressed in any of the individuals, whilst 6,281 (13.3%) are detected as expressed in all individuals. (DOCX) [file pone.0035430.s002.docx]

**Figure S2**


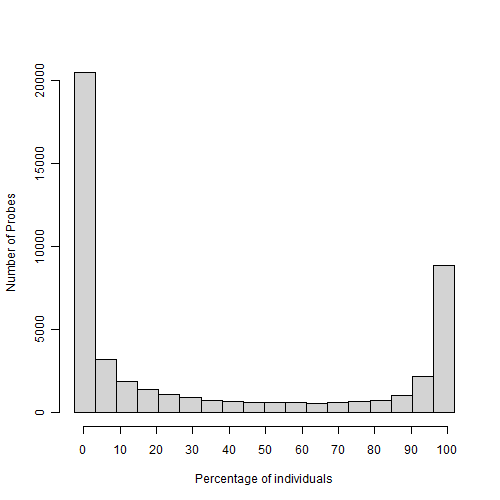


**Figure S2 |** The distribution of the number of probes detected as expressed in stage II. A total of 47,323 are measured on the Illumina HT-12 v4.0 chip, of these 5,364 (11.3%) are not detected as expressed in any of the individuals, whilst 6,281 (13.3%) are detected as expressed in all individuals.
