# Supplementary material for: The Brisbane Systems Genetics Study: Genetical Genomics Meets Complex Trait Genetics
Source: PLoS One. 2012 Apr 26;7(4):e35430. doi: 10.1371/journal.pone.0035430 (PMC3338511; doi:10.1371/journal.pone.0035430)
Supplement: Figure S3 — A Principle Component Analysis (PCA) of 16 global populations and the individuals collected in this study. Principal Component one (PC1) and two (PC2) values were derived from approximately 280,000 autosomal markers. Populations samples marked with *1 were collected as part of the HapHap3 project [1] and *2 as part of the GenomEUtwin project [2]. ASW *1 African Americans, CEU *1 European Americans, CHB *1 Han Chinese, CHD *1 Chinese, GIH *1 Guajarati-Indians, JPT *1 Japanese, LWK *1 Luhya Kenyans, MEX *1 Mexicans, MKK *1 Maasai Kenyans, TSI *1 Italians, TRI *1 Yorubans Nigeria, DEN *2 Danish, FIN *2 Finish, NET *2 Dutch, SWE *2 Swedish, UK *2 British. (DOCX) [file pone.0035430.s003.docx]

**Figure S3**
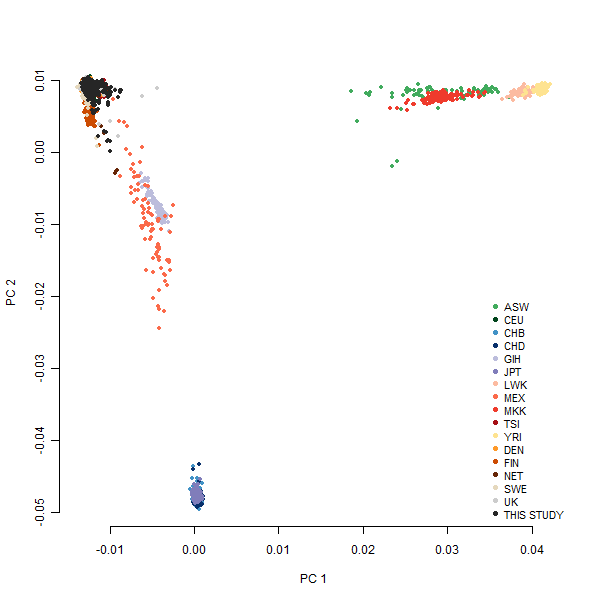


**Figure S3 |** A Principle Component Analysis (PCA) of 16 global populations and the individuals collected in this study. Principal Component one (PC1) and two (PC2) values were derived from approximately 280,000 autosomal markers. Populations samples marked with *1 were collected as part of the HapHap3 project [1] and *2 as part of the GenomEUtwin project [2]. **ASW**^*1^ African Americans, **CEU**^*1^ European Americans, **CHB**^*1^ Han Chinese, **CHD**^*1^ Chinese, **GIH**^*1^ Guajarati-Indians, **JPT**^*1^ Japanese, **LWK**^*1^ Luhya Kenyans, **MEX**^*1^ Mexicans, **MKK**^*1^ Maasai Kenyans, **TSI**^*1^ Italians, **TRI**^*1^ Yorubans Nigeria, **DEN**^*2^ Danish, **FIN**^*2^ Finish, **NET**^*2^ Dutch, **SWE**^*2^ Swedish, **UK**^*2^ British.

**References**

1. International HapMap 3 Consortium. 2010. Integrating common and rare genetic variation in diverse human populations. *Nature* **467**(7311): 52-58.

2. Peltonen L. 2003. GenomEUtwin: a strategy to identify genetic influences on health and disease. *Twin Res* **6**(5): 354-360.
